# Diethyl 2-{[(5-oxo-5*H*-thio­chromeno[2,3-*b*]pyridin-7-yl)amino]­methyl­idene}propane­dioate

**DOI:** 10.1107/S1600536811016291

**Published:** 2011-05-07

**Authors:** Muhammad Naeem Khan, M. Nawaz Tahir, Misbahul Ain Khan, Munawar Ali Munawar, Abdul Qayyum Ather

**Affiliations:** a Department of Chemistry, Islamia University, Bahawalpur, Pakistan; b Applied Chemistry Research Center, PCSIR Laboratories complex, Lahore 54600, Pakistan; c University of Sargodha, Department of Physics, Sargodha, Pakistan; d Institute of Chemistry, University of the Punjab, Lahore, Pakistan

## Abstract

In the title compound, C_15_H_14_O_2_S, the three fused rings are roughly coplanar, the largest deviation from the mean plane being 0.1285 (13) Å for the S atom. An intra­molecular N—H⋯O hydrogen bond generates an *S*6 ring. In the crystal, inter­molecular C—H⋯O hydrogen bonds form *R*_2_^2^(14), *R_2_* ^2^(13) and *R*_3_^2^(17) ring motifs, building a layer parallel to (100).

## Related literature

For related structures, see: Khan *et al.* (2008**a* ▶,b* ▶); Lokaj *et al.* (1994 ▶); Lynch & McClenaghan (2003 ▶); For graph-set notation, see: Etter *et al.* (1990 ▶); Bernstein *et al.* (1995 ▶).
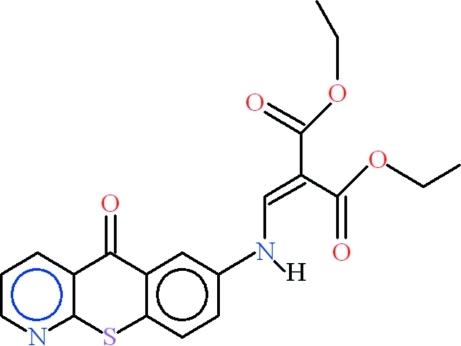


## Experimental

### 

#### Crystal data

C_20_H_18_N_2_O_5_S
*M* *_r_* = 398.42
Monoclinic, 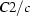

*a* = 13.8013 (7) Å
*b* = 7.5180 (3) Å
*c* = 36.2743 (15) Å
β = 94.330 (3)°
*V* = 3753.0 (3) Å^3^
*Z* = 8
Mo *K*α radiation
μ = 0.21 mm^−1^
*T* = 296 K
0.35 × 0.18 × 0.15 mm

#### Data collection

Bruker Kappa APEXII CCD diffractometer
Absorption correction: multi-scan (*SADABS*; Bruker, 2005 ▶) *T*_min_ = 0.968, *T*_max_ = 0.985
13274 measured reflections
3314 independent reflections
1776 reflections with *I* > 2σ(*I*)
*R*_int_ = 0.077

#### Refinement

*R*[*F* ^2^ > 2σ(*F* ^2^)] = 0.054
*wR*(*F* ^2^) = 0.130
*S* = 0.97
3314 reflections
269 parameters
10 restraints
H-atom parameters constrained
Δρ_max_ = 0.20 e Å^−3^
Δρ_min_ = −0.18 e Å^−3^

### 

Data collection: *APEX2* (Bruker, 2009 ▶); cell refinement: *SAINT* (Bruker, 2009 ▶); data reduction: *SAINT*; program(s) used to solve structure: *SHELXS97* (Sheldrick, 2008 ▶); program(s) used to refine structure: *SHELXL97* (Sheldrick, 2008 ▶); molecular graphics: *ORTEPIII* (Burnett & Johnson, 1996 ▶), *ORTEP-3 for Windows* (Farrugia, 1997 ▶) and *PLATON* (Spek, 2009 ▶)’; software used to prepare material for publication: *SHELXL97*.

## Supplementary Material

Crystal structure: contains datablocks global, I. DOI: 10.1107/S1600536811016291/dn2678sup1.cif

Structure factors: contains datablocks I. DOI: 10.1107/S1600536811016291/dn2678Isup2.hkl

Supplementary material file. DOI: 10.1107/S1600536811016291/dn2678Isup3.cml

Additional supplementary materials:  crystallographic information; 3D view; checkCIF report

## Figures and Tables

**Table 1:** Hydrogen-bond geometry (Å, °)

*D*—H⋯*A*	*D*—H	H⋯*A*	*D*⋯*A*	*D*—H⋯*A*
N2—H2⋯O3	0.86	2.06	2.683 (3)	129
C11—H11⋯O5^i^	0.93	2.49	3.403 (4)	167
C12—H12⋯O1^i^	0.93	2.45	3.322 (4)	157
C17*A*—H17*C*⋯O3^ii^	0.96	2.58	3.516 (8)	165
C19*A*—H19*B*⋯O3^iii^	0.97	2.47	3.231 (11)	135

Symmetry codes: (i) 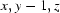; (ii) 
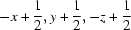
; (iii) 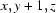.

**Fig. 1.**
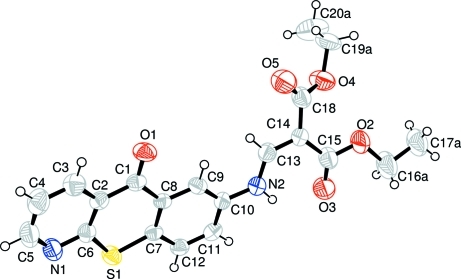
View of the title compound with the atom numbering scheme. The thermal displacements are drawn at the 50% probability level. H-atoms are shown as small spheres of arbitrary radii. The disordered miniority ethyl groups are omitted for clarity.

**Fig. 2.**
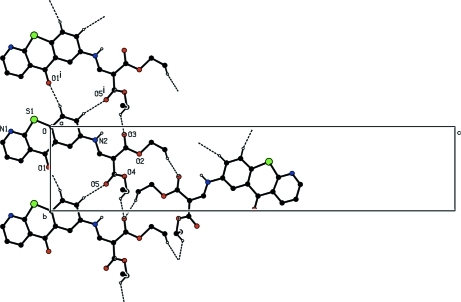
Partial packing view showing the formation of different ring motifs through C-H···O hydrogen bonds. Only the major component of the disordered moieties are represented and H atoms not involved in hydrogen bondings have been omitted for clarity. [Symmetry code: (i) x, y-1, z.]

## supplementary crystallographic information

### Comment

We reported the crystal structures of (II) *i.e.* 7-nitro-5*H*-thiochromeno[2,3-*b*]pyridin-5-one (Khan *et al.*, 2008*a*) and (III) *i.e.* 5*H*-thiochromeno[2,3-*b*]pyridin-5-one (Khan *et al.*, 2008*b*). The title compound (Fig. 1) is in continuation of our work to synthesize the derivatives of 5*H*-thiochromeno[2,3-*b*]pyridin-5-one. The crystal structures of (IV) *i.e.*, diethyl 2-(2,3-diphenylquinoxalin-6-ylaminomethylene)malonate (Lokaj *et al.*, 1994) and (V) *i.e.*, diethyl (4-*tert*-butyl-1,3-thiazol-2-ylaminomethylene)malonate (Lynch & McClenaghan, 2003) have been published. Both contain the diethyl (aminomethylidene)propanedioate moiety which is also present in (I).

In (I), the central heterocyclic ring A (C1/C2/C6/S1/C7/C8), the pyridinic group B (C2/C3/C4/C5/N1/C6) and benzene ring C (C7—C12) are planar with r. m. s. deviation of 0.0388, 0.0053 and 0.0049 Å, respectively. The carbonyl O-atom is at a distance of 0.1532 (35) Å from its parent ring A. The dihedral angle between A/B, A/C and B/C is 3.68 (12), 3.50 (9) and 7.19 (12)°, respectively. A strong intramolecular H-bonding of N—H···O type completes an S(6) (Etter *et al.*, 1990; Bernstein *et al.*, 1995) ring motif. Intermolecular C—H···O hydrogen bonds complete *R*_2_^2^(14), *R_2_*^2^(13) and *R*_3_^2^(17) ring motifs building a layer parallel to the (1 0 0) plane (Table 1, Fig 2).

### Experimental

Diethyl (ethoxymethylene)malonate (0.473 g, 2.1 mmol), 7-amino-5*H*-thiochromeno[2,3-*b*]pyridin-5-one (500 mg, 2.1 mmol) and 8.0 ml of ethyl alcohol was heated under reflux on water bath for 3 h. Completion of reaction was monitored by TLC. The product precipitated by the addition of n-hexane was filtered, washed, dried and recrystallized from chloroform to give the yellow needles of (I).

### Refinement

The diethyl groups are disordered over two set of sites with occupancy ratio of 0.75:0.25 and 0.53:0.47. The disordered poitions were refined using O-C and C-C bond restraints to maintain chemically reasonable geometry. The disordered groups were refined anistropically with however equal thermal parameters for the C-atoms.

The H-atoms were positioned geometrically (N—H = 0.86, C–H = 0.93–0.97 Å) and treated as riding with *U*_iso_(H) = *xU*_eq_(C, O), where *x* = 1.5 for methyl and *x* = 1.2 for aryl H-atoms.

### Crystal data

**Table:**

C_20_H_18_N_2_O_5_S	*F*(000) = 1664
*M**_r_* = 398.42	*D*_x_ = 1.410 Mg m^−^^3^
Monoclinic, *C*2/*c*	Mo *K*α radiation, λ = 0.71073 Å
Hall symbol: -C 2yc	Cell parameters from 1789 reflections
*a* = 13.8013 (7) Å	θ = 2.3–25.3°
*b* = 7.5180 (3) Å	µ = 0.21 mm^−^^1^
*c* = 36.2743 (15) Å	*T* = 296 K
β = 94.330 (3)°	Needle, yellow
*V* = 3753.0 (3) Å^3^	0.35 × 0.18 × 0.15 mm
*Z* = 8	

### Data collection

**Table:**

Bruker Kappa APEXII CCD diffractometer	3314 independent reflections
Radiation source: fine-focus sealed tube	1776 reflections with *I* > 2σ(*I*)
graphite	*R*_int_ = 0.077
Detector resolution: 8.10 pixels mm^-1^	θ_max_ = 25.0°, θ_min_ = 2.3°
ω scans	*h* = −16→16
Absorption correction: multi-scan (*SADABS*; Bruker, 2005)	*k* = 0→8
*T*_min_ = 0.968, *T*_max_ = 0.985	*l* = 0→42
13274 measured reflections	

### Refinement

**Table:**

Refinement on *F*^2^	Primary atom site location: structure-invariant direct methods
Least-squares matrix: full	Secondary atom site location: difference Fourier map
*R*[*F*^2^ > 2σ(*F*^2^)] = 0.054	Hydrogen site location: inferred from neighbouring sites
*wR*(*F*^2^) = 0.130	H-atom parameters constrained
*S* = 0.97	*w* = 1/[σ^2^(*F*_o_^2^) + (0.0533*P*)^2^] where *P* = (*F*_o_^2^ + 2*F*_c_^2^)/3
3314 reflections	(Δ/σ)_max_ = 0.001
269 parameters	Δρ_max_ = 0.20 e Å^−^^3^
10 restraints	Δρ_min_ = −0.18 e Å^−^^3^

### Special details

**Table:**

Geometry. All esds (except the esd in the dihedral angle between two l.s. planes) are estimated using the full covariance matrix. The cell esds are taken into account individually in the estimation of esds in distances, angles and torsion angles; correlations between esds in cell parameters are only used when they are defined by crystal symmetry. An approximate (isotropic) treatment of cell esds is used for estimating esds involving l.s. planes.
Refinement. Refinement of *F*^2^ against ALL reflections. The weighted *R*-factor *wR* and goodness of fit *S* are based on *F*^2^, conventional *R*-factors *R* are based on *F*, with *F* set to zero for negative *F*^2^. The threshold expression of *F*^2^ > σ(*F*^2^) is used only for calculating *R*-factors(gt) *etc*. and is not relevant to the choice of reflections for refinement. *R*-factors based on *F*^2^ are statistically about twice as large as those based on *F*, and *R*- factors based on ALL data will be even larger.

### Fractional atomic coordinates and isotropic or equivalent isotropic displacement parameters (Å^2^)

**Table:**

	*x*	*y*	*z*	*U*_iso_*/*U*_eq_	Occ. (<1)
S1	0.10364 (6)	−0.08366 (11)	−0.04024 (2)	0.0529 (3)	
O1	0.11162 (17)	0.4807 (3)	−0.00428 (6)	0.0604 (7)	
O2	0.3194 (2)	0.3243 (3)	0.21973 (6)	0.0911 (9)	
C16A	0.3217 (7)	0.1867 (9)	0.24963 (15)	0.1062 (19)	0.75
H16A	0.2563	0.1492	0.2540	0.127*	0.75
H16B	0.3585	0.0834	0.2428	0.127*	0.75
C17A	0.3688 (6)	0.2726 (9)	0.28278 (13)	0.1062 (19)	0.75
H17A	0.4333	0.3086	0.2780	0.159*	0.75
H17B	0.3718	0.1901	0.3030	0.159*	0.75
H17C	0.3318	0.3751	0.2890	0.159*	0.75
C16B	0.2928 (14)	0.259 (3)	0.2569 (5)	0.122 (7)	0.25
H16C	0.2441	0.1659	0.2556	0.147*	0.25
H16D	0.2762	0.3534	0.2736	0.147*	0.25
C17B	0.3965 (14)	0.192 (3)	0.2636 (8)	0.122 (7)	0.25
H17D	0.4131	0.1221	0.2429	0.184*	0.25
H17E	0.4019	0.1198	0.2855	0.184*	0.25
H17F	0.4401	0.2912	0.2668	0.184*	0.25
O3	0.2852 (2)	0.0944 (4)	0.18250 (6)	0.0853 (9)	
O4	0.40074 (19)	0.5902 (3)	0.18304 (7)	0.0793 (8)	
C19A	0.4388 (7)	0.7695 (14)	0.1889 (4)	0.105 (3)	0.53
H19A	0.4366	0.8060	0.2145	0.126*	0.53
H19B	0.4031	0.8548	0.1730	0.126*	0.53
C20A	0.5401 (7)	0.7499 (13)	0.1787 (3)	0.105 (3)	0.53
H20A	0.5726	0.6611	0.1941	0.157*	0.53
H20B	0.5734	0.8614	0.1822	0.157*	0.53
H20C	0.5399	0.7146	0.1533	0.157*	0.53
C19B	0.4500 (8)	0.7644 (17)	0.1791 (5)	0.108 (4)	0.47
H19C	0.4098	0.8599	0.1874	0.130*	0.47
H19D	0.4615	0.7854	0.1534	0.130*	0.47
C20B	0.5450 (7)	0.7572 (16)	0.2024 (3)	0.108 (4)	0.47
H20D	0.5325	0.7451	0.2280	0.163*	0.47
H20E	0.5808	0.8647	0.1990	0.163*	0.47
H20F	0.5822	0.6571	0.1950	0.163*	0.47
O5	0.2939 (2)	0.6807 (4)	0.13788 (7)	0.0874 (9)	
N1	0.0155 (2)	0.0557 (4)	−0.09743 (7)	0.0591 (8)	
N2	0.23819 (19)	0.1593 (4)	0.11064 (7)	0.0544 (8)	
H2	0.2432	0.0765	0.1270	0.065*	
C1	0.1051 (2)	0.3242 (5)	−0.01327 (8)	0.0440 (8)	
C2	0.0601 (2)	0.2734 (4)	−0.05003 (8)	0.0446 (8)	
C3	0.0182 (2)	0.4052 (5)	−0.07279 (9)	0.0618 (10)	
H3	0.0195	0.5229	−0.0649	0.074*	
C4	−0.0251 (3)	0.3623 (6)	−0.10695 (10)	0.0737 (12)	
H4	−0.0542	0.4491	−0.1223	0.088*	
C5	−0.0242 (3)	0.1861 (6)	−0.11782 (9)	0.0719 (11)	
H5	−0.0533	0.1574	−0.1410	0.086*	
C6	0.0558 (2)	0.1015 (4)	−0.06379 (8)	0.0460 (8)	
C7	0.1410 (2)	0.0021 (4)	0.00313 (8)	0.0407 (8)	
C8	0.1395 (2)	0.1820 (4)	0.01247 (8)	0.0390 (8)	
C9	0.1711 (2)	0.2333 (4)	0.04837 (8)	0.0452 (8)	
H9	0.1691	0.3528	0.0549	0.054*	
C10	0.2052 (2)	0.1102 (5)	0.07425 (8)	0.0448 (8)	
C11	0.2065 (2)	−0.0693 (4)	0.06457 (8)	0.0507 (9)	
H11	0.2294	−0.1534	0.0819	0.061*	
C12	0.1743 (2)	−0.1219 (4)	0.02973 (8)	0.0468 (8)	
H12	0.1746	−0.2421	0.0237	0.056*	
C13	0.2620 (2)	0.3227 (5)	0.12156 (8)	0.0525 (9)	
H13	0.2555	0.4106	0.1035	0.063*	
C14	0.2949 (2)	0.3776 (4)	0.15601 (8)	0.0503 (9)	
C15	0.3008 (3)	0.2515 (6)	0.18640 (9)	0.0634 (10)	
C18	0.3268 (3)	0.5646 (5)	0.15800 (9)	0.0608 (10)	

### Atomic displacement parameters (Å^2^)

**Table:**

	*U*^11^	*U*^22^	*U*^33^	*U*^12^	*U*^13^	*U*^23^
S1	0.0609 (6)	0.0445 (6)	0.0529 (5)	0.0014 (5)	0.0003 (4)	−0.0085 (4)
O1	0.0728 (18)	0.0357 (15)	0.0714 (15)	0.0011 (13)	−0.0036 (12)	−0.0025 (12)
O2	0.150 (3)	0.077 (2)	0.0444 (14)	−0.0195 (18)	−0.0106 (15)	0.0048 (13)
C16A	0.175 (6)	0.088 (4)	0.050 (2)	0.010 (4)	−0.023 (3)	0.004 (2)
C17A	0.175 (6)	0.088 (4)	0.050 (2)	0.010 (4)	−0.023 (3)	0.004 (2)
C16B	0.20 (2)	0.056 (13)	0.107 (13)	−0.034 (13)	0.004 (15)	0.009 (9)
C17B	0.20 (2)	0.056 (13)	0.107 (13)	−0.034 (13)	0.004 (15)	0.009 (9)
O3	0.135 (3)	0.0529 (19)	0.0666 (16)	−0.0019 (18)	0.0001 (15)	0.0044 (14)
O4	0.085 (2)	0.0608 (19)	0.0880 (18)	−0.0109 (15)	−0.0233 (15)	−0.0001 (14)
C19A	0.127 (9)	0.055 (5)	0.129 (7)	−0.022 (5)	−0.010 (6)	0.006 (4)
C20A	0.127 (9)	0.055 (5)	0.129 (7)	−0.022 (5)	−0.010 (6)	0.006 (4)
C19B	0.080 (8)	0.102 (8)	0.138 (8)	−0.009 (5)	−0.026 (5)	−0.044 (6)
C20B	0.080 (8)	0.102 (8)	0.138 (8)	−0.009 (5)	−0.026 (5)	−0.044 (6)
O5	0.115 (2)	0.063 (2)	0.0792 (18)	0.0021 (16)	−0.0280 (16)	0.0106 (15)
N1	0.058 (2)	0.072 (2)	0.0474 (17)	−0.0035 (16)	0.0008 (14)	−0.0064 (15)
N2	0.065 (2)	0.053 (2)	0.0452 (16)	−0.0068 (16)	0.0044 (13)	0.0012 (13)
C1	0.034 (2)	0.044 (2)	0.054 (2)	−0.0031 (17)	0.0088 (15)	0.0006 (17)
C2	0.038 (2)	0.046 (2)	0.0496 (19)	0.0001 (16)	0.0032 (15)	0.0073 (16)
C3	0.060 (2)	0.059 (3)	0.065 (2)	0.003 (2)	0.0019 (19)	0.003 (2)
C4	0.075 (3)	0.081 (3)	0.063 (3)	0.007 (2)	−0.008 (2)	0.016 (2)
C5	0.075 (3)	0.091 (4)	0.049 (2)	−0.001 (3)	−0.0056 (19)	0.000 (2)
C6	0.039 (2)	0.054 (2)	0.0446 (18)	−0.0004 (17)	0.0057 (15)	0.0009 (17)
C7	0.035 (2)	0.039 (2)	0.0479 (18)	−0.0044 (16)	0.0062 (15)	0.0002 (15)
C8	0.0351 (19)	0.037 (2)	0.0452 (18)	−0.0044 (15)	0.0061 (14)	−0.0006 (15)
C9	0.044 (2)	0.042 (2)	0.0507 (19)	−0.0033 (16)	0.0073 (16)	−0.0075 (16)
C10	0.047 (2)	0.050 (2)	0.0386 (17)	−0.0050 (17)	0.0066 (14)	0.0010 (16)
C11	0.053 (2)	0.046 (2)	0.053 (2)	−0.0014 (18)	0.0071 (17)	0.0101 (17)
C12	0.051 (2)	0.037 (2)	0.052 (2)	−0.0022 (16)	0.0077 (16)	−0.0007 (15)
C13	0.051 (2)	0.060 (3)	0.047 (2)	−0.0038 (19)	0.0060 (16)	0.0018 (17)
C14	0.059 (2)	0.049 (2)	0.0430 (18)	−0.0047 (18)	0.0061 (16)	0.0007 (16)
C15	0.072 (3)	0.066 (3)	0.051 (2)	0.003 (2)	−0.0017 (19)	−0.005 (2)
C18	0.067 (3)	0.064 (3)	0.050 (2)	0.001 (2)	−0.0044 (19)	−0.0032 (19)

### Geometric parameters (Å, °)

**Table:**

S1—C6	1.737 (3)	C20B—H20D	0.9600
S1—C7	1.742 (3)	C20B—H20E	0.9600
O1—C1	1.222 (3)	C20B—H20F	0.9600
O2—C15	1.334 (4)	O5—C18	1.204 (4)
O2—C16A	1.497 (6)	N1—C5	1.321 (4)
O2—C16B	1.506 (14)	N1—C6	1.347 (4)
C16A—C17A	1.472 (7)	N2—C13	1.324 (4)
C16A—H16A	0.9700	N2—C10	1.413 (4)
C16A—H16B	0.9700	N2—H2	0.8600
C17A—H17A	0.9600	C1—C8	1.474 (4)
C17A—H17B	0.9600	C1—C2	1.478 (4)
C17A—H17C	0.9600	C2—C6	1.385 (4)
C16B—C17B	1.520 (10)	C2—C3	1.388 (4)
C16B—H16C	0.9700	C3—C4	1.373 (4)
C16B—H16D	0.9700	C3—H3	0.9300
C17B—H17D	0.9600	C4—C5	1.382 (5)
C17B—H17E	0.9600	C4—H4	0.9300
C17B—H17F	0.9600	C5—H5	0.9300
O3—C15	1.206 (4)	C7—C12	1.395 (4)
O4—C18	1.328 (4)	C7—C8	1.395 (4)
O4—C19A	1.457 (10)	C8—C9	1.396 (4)
O4—C19B	1.487 (12)	C9—C10	1.375 (4)
C19A—C20A	1.480 (9)	C9—H9	0.9300
C19A—H19A	0.9700	C10—C11	1.395 (4)
C19A—H19B	0.9700	C11—C12	1.366 (4)
C20A—H20A	0.9600	C11—H11	0.9300
C20A—H20B	0.9600	C12—H12	0.9300
C20A—H20C	0.9600	C13—C14	1.361 (4)
C19B—C20B	1.506 (10)	C13—H13	0.9300
C19B—H19C	0.9700	C14—C15	1.452 (4)
C19B—H19D	0.9700	C14—C18	1.473 (4)
			
C6—S1—C7	102.90 (15)	C6—C2—C1	124.7 (3)
C15—O2—C16A	111.4 (4)	C3—C2—C1	118.7 (3)
C15—O2—C16B	129.4 (11)	C4—C3—C2	120.2 (3)
C16A—O2—C16B	28.4 (8)	C4—C3—H3	119.9
C17A—C16A—O2	105.8 (5)	C2—C3—H3	119.9
C17A—C16A—H16A	110.6	C3—C4—C5	118.0 (4)
O2—C16A—H16A	110.6	C3—C4—H4	121.0
C17A—C16A—H16B	110.6	C5—C4—H4	121.0
O2—C16A—H16B	110.6	N1—C5—C4	124.3 (4)
H16A—C16A—H16B	108.7	N1—C5—H5	117.9
O2—C16B—C17B	87.8 (14)	C4—C5—H5	117.9
O2—C16B—H16C	114.0	N1—C6—C2	124.6 (3)
C17B—C16B—H16C	114.0	N1—C6—S1	110.9 (3)
O2—C16B—H16D	114.0	C2—C6—S1	124.5 (2)
C17B—C16B—H16D	114.0	C12—C7—C8	119.4 (3)
H16C—C16B—H16D	111.2	C12—C7—S1	115.9 (2)
C16B—C17B—H17D	109.5	C8—C7—S1	124.7 (2)
C16B—C17B—H17E	109.5	C7—C8—C9	119.0 (3)
H17D—C17B—H17E	109.5	C7—C8—C1	124.0 (3)
C16B—C17B—H17F	109.5	C9—C8—C1	117.0 (3)
H17D—C17B—H17F	109.5	C10—C9—C8	121.2 (3)
H17E—C17B—H17F	109.5	C10—C9—H9	119.4
C18—O4—C19A	118.9 (6)	C8—C9—H9	119.4
C18—O4—C19B	113.3 (6)	C9—C10—C11	119.3 (3)
C19A—O4—C19B	15.6 (11)	C9—C10—N2	122.1 (3)
O4—C19A—C20A	102.0 (8)	C11—C10—N2	118.6 (3)
O4—C19A—H19A	111.4	C12—C11—C10	120.2 (3)
C20A—C19A—H19A	111.4	C12—C11—H11	119.9
O4—C19A—H19B	111.4	C10—C11—H11	119.9
C20A—C19A—H19B	111.4	C11—C12—C7	120.9 (3)
H19A—C19A—H19B	109.2	C11—C12—H12	119.5
O4—C19B—C20B	107.4 (10)	C7—C12—H12	119.5
O4—C19B—H19C	110.2	N2—C13—C14	127.9 (3)
C20B—C19B—H19C	110.2	N2—C13—H13	116.1
O4—C19B—H19D	110.2	C14—C13—H13	116.1
C20B—C19B—H19D	110.2	C13—C14—C15	119.7 (3)
H19C—C19B—H19D	108.5	C13—C14—C18	114.3 (3)
C5—N1—C6	116.3 (3)	C15—C14—C18	125.9 (3)
C13—N2—C10	125.3 (3)	O3—C15—O2	121.9 (3)
C13—N2—H2	117.4	O3—C15—C14	123.4 (3)
C10—N2—H2	117.4	O2—C15—C14	114.6 (4)
O1—C1—C8	121.0 (3)	O5—C18—O4	123.0 (4)
O1—C1—C2	120.4 (3)	O5—C18—C14	124.4 (4)
C8—C1—C2	118.5 (3)	O4—C18—C14	112.5 (3)
C6—C2—C3	116.6 (3)		
			
C15—O2—C16A—C17A	164.6 (6)	O1—C1—C8—C9	5.8 (4)
C16B—O2—C16A—C17A	−60.2 (19)	C2—C1—C8—C9	−172.7 (2)
C15—O2—C16B—C17B	101.0 (17)	C7—C8—C9—C10	1.2 (4)
C16A—O2—C16B—C17B	42.9 (14)	C1—C8—C9—C10	−179.5 (3)
C18—O4—C19A—C20A	−118.4 (9)	C8—C9—C10—C11	−1.1 (4)
C19B—O4—C19A—C20A	−46 (3)	C8—C9—C10—N2	179.5 (3)
C18—O4—C19B—C20B	−166.4 (9)	C13—N2—C10—C9	−16.8 (5)
C19A—O4—C19B—C20B	79 (3)	C13—N2—C10—C11	163.8 (3)
O1—C1—C2—C6	174.9 (3)	C9—C10—C11—C12	0.0 (4)
C8—C1—C2—C6	−6.5 (4)	N2—C10—C11—C12	179.4 (3)
O1—C1—C2—C3	−5.4 (4)	C10—C11—C12—C7	1.0 (4)
C8—C1—C2—C3	173.2 (3)	C8—C7—C12—C11	−0.9 (4)
C6—C2—C3—C4	0.4 (5)	S1—C7—C12—C11	179.2 (2)
C1—C2—C3—C4	−179.3 (3)	C10—N2—C13—C14	−179.4 (3)
C2—C3—C4—C5	−0.9 (5)	N2—C13—C14—C15	−4.2 (5)
C6—N1—C5—C4	0.9 (5)	N2—C13—C14—C18	172.5 (3)
C3—C4—C5—N1	0.2 (6)	C16A—O2—C15—O3	2.2 (6)
C5—N1—C6—C2	−1.5 (5)	C16B—O2—C15—O3	−23.5 (11)
C5—N1—C6—S1	179.4 (2)	C16A—O2—C15—C14	178.4 (5)
C3—C2—C6—N1	0.9 (4)	C16B—O2—C15—C14	152.7 (10)
C1—C2—C6—N1	−179.4 (3)	C13—C14—C15—O3	6.0 (5)
C3—C2—C6—S1	179.8 (2)	C18—C14—C15—O3	−170.3 (4)
C1—C2—C6—S1	−0.5 (4)	C13—C14—C15—O2	−170.2 (3)
C7—S1—C6—N1	−174.8 (2)	C18—C14—C15—O2	13.5 (5)
C7—S1—C6—C2	6.2 (3)	C19A—O4—C18—O5	5.6 (8)
C6—S1—C7—C12	173.8 (2)	C19B—O4—C18—O5	−10.6 (8)
C6—S1—C7—C8	−6.1 (3)	C19A—O4—C18—C14	−178.4 (6)
C12—C7—C8—C9	−0.2 (4)	C19B—O4—C18—C14	165.4 (7)
S1—C7—C8—C9	179.7 (2)	C13—C14—C18—O5	29.7 (5)
C12—C7—C8—C1	−179.4 (3)	C15—C14—C18—O5	−153.8 (4)
S1—C7—C8—C1	0.5 (4)	C13—C14—C18—O4	−146.2 (3)
O1—C1—C8—C7	−175.0 (3)	C15—C14—C18—O4	30.3 (5)
C2—C1—C8—C7	6.5 (4)		

### Hydrogen-bond geometry (Å, °)

**Table:**

*D*—H···*A*	*D*—H	H···*A*	*D*···*A*	*D*—H···*A*
N2—H2···O3	0.86	2.06	2.683 (3)	129
C11—H11···O5^i^	0.93	2.49	3.403 (4)	167
C12—H12···O1^i^	0.93	2.45	3.322 (4)	157
C17A—H17C···O3^ii^	0.96	2.58	3.516 (8)	165
C19A—H19B···O3^iii^	0.97	2.47	3.231 (11)	135

Symmetry codes: (i) *x*, *y*−1, *z*; (ii) −*x*+1/2, *y*+1/2, −*z*+1/2; (iii) *x*, *y*+1, *z*.
